# Blind Calibration of Environmental Acoustics Measurements Using Smartphones

**DOI:** 10.3390/s24041255

**Published:** 2024-02-16

**Authors:** Ayoub Boumchich, Judicaël Picaut, Pierre Aumond, Arnaud Can, Erwan Bocher

**Affiliations:** 1 UMRAE, CEREMA, Univ Gustave Eiffel, F-44344 Bouguenais, France; ayoub.boumchich@univ-eiffel.fr (A.B.); pierre.aumond@univ-eiffel.fr (P.A.); arnaud.can@univ-eiffel.fr (A.C.); 2 Lab-STICC, UMR 6285, CNRS, Université Bretagne Sud, F-56000 Vannes, France; erwan.bocher@cnrs.fr

**Keywords:** environmental noise, noise mapping, smartphone application, calibration

## Abstract

Environmental noise control is a major health and social issue. Numerous environmental policies require local authorities to draw up noise maps to establish an inventory of the noise environment and then propose action plans to improve its quality. In general, these maps are produced using numerical simulations, which may not be sufficiently representative, for example, concerning the temporal dynamics of noise levels. Acoustic sensor measurements are also insufficient in terms of spatial coverage. More recently, an alternative approach has been proposed, consisting of using citizens as data producers by using smartphones as tools of geo-localized acoustic measurement. However, a lack of calibration of smartphones can generate a significant bias in the results obtained. Against the classical metrological principle that would aim to calibrate any sensor beforehand for physical measurement, some have proposed mass calibration procedures called “blind calibration”. The method is based on the crossing of sensors in the same area at the same time, which are therefore supposed to observe the same phenomenon (i.e., measure the same value). The multiple crossings of a large number of sensors at the scale of a territory and the analysis of the relationships between sensors allow for the calibration of the set of sensors. In this article, we propose to adapt a blind calibration method to data from the NoiseCapture smartphone application. The method’s behavior is then tested on NoiseCapture datasets for which information on the calibration values of some smartphones is already available.

## 1. Introduction

Managing environmental noise, particularly in urban areas, is a major health and social issue. Numerous environmental policies encourage local authorities to produce noise maps of their territory with the aim of establishing an inventory of the noise environment and then proposing action plans to improve its quality. This is the case, for example, with the European directive 2002/49/EC [1] relating to the assessment and management of environmental noise.

The production of noise maps remains the most widely used tool when considering environmental policies. In general, these maps are produced using simulations based on calculation models requiring traffic data for the calculation of acoustic emission and spatial data for the modeling of acoustic propagation. Because access to these data is sometimes complicated, and their quality is sometimes questionable, the result of the simulations only partially reflects the existing state of the sound environment. Conversely, the use of acoustic sensors arranged within noise observatories gives a more detailed and realistic image of the noise environment of an area, but the insufficient number of sensors available does not allow for covering the whole territory and producing a detailed noise map [2].

The densification of sensors through the deployment of low-cost sensor networks is an interesting alternative, but the network thus produced may prove difficult to maintain in the long term. Although several experiments have already taken place, to our knowledge, there is no functional network of this type that can produce noise maps.

Another alternative is for citizens to become data producers themselves, using smartphones as measuring instruments, as part of a participative or crowd-sourcing approach. On this subject, since the pioneering work in the early 2010s [3,4,5], many studies have been conducted [6,7], notably on the quality of acoustic measurements produced with a smartphone, as well as on the implementation of a participatory approach to collect data on a large scale and over the long term. Among these approaches, the one based on the NoiseCapture application, which was developed in our laboratory, is the most advanced today [8]. Since the application was released in 2017 (for Android smartphones only), a considerable amount of data has been collected worldwide [9]. Analysis of the data revealed a wide range in the quality of the noise indicators collected due to the measurement protocol and, in particular, the lack of acoustic calibration of the smartphones in most cases. A lack of calibration, or even a bad calibration, can indeed generate a significant bias in the measurement results. The realization of a calibration in the state of the art, from a reference device (for example, an acoustic calibrator), would normally constitute a prerequisite for the realization of measurements, but the access to such reference devices by any citizen makes this procedure difficult to apply in practice. The proportion of calibrated smartphones in the totality of collected data is then very low, making its use for the production of noise maps more difficult.

In contrast to the classical metrological principle of calibrating any sensor for physical measurement, others have proposed so-called “blind” mass calibration procedures. The method is based on the crossing of sensors in the same area, at the same time, which are therefore supposed to observe the same phenomenon (i.e., to measure the same value). The repetition of these crossings of a large number of sensors at the scale of a territory, and the analysis of the relations between sensors allow, in theory, to calibrate all the sensors. This type of blind calibration seems particularly interesting for data such as those collected by NoiseCapture, especially in urban areas, where several sensors can cross each other in the same area at equivalent time periods.

In this paper, we propose to implement a blind calibration method for uncalibrated mobile noise measurements. The approach itself is not novel, since blind calibration has already been applied in other fields, but its application to a database consisting of geo-localized acoustic measurements is, in our opinion, a major step forward, calling into question the need to calibrate each smartphone individually. In the present work, this approach is applied on NoiseCapture data, but it could be generalized for any equivalent dataset. In the present case, we have exploited the NoiseCapture dataset for the 2017–2020 period available for download [10] as well as more recent additional data obtained by connecting to the online database [11]. It is important to mention at this point that all data collected with the NoiseCapture application are totally anonymous. The application fully respects users’ privacy [12].

The method, described in Section 2, is based on modeling the relationships between sensors, which can be written in matrix form, and which can then be solved as a linear algebra problem. The behavior of the method, as well as a modified model, is then tested on NoiseCapture datasets for which information on the calibration values of some smartphones is available (Section 3). Finally, as an experiment, the method is applied to the dataset of the City of Rezé in France, allowing the production of a “calibrated” noise map based on the collected raw data (Section 3.5). Section 4 concludes on the next challenges to deploy this method on a large variety of territories.

## 2. Methodology

### 2.1. The Problem of the Acoustic Calibration of Smartphones on a Large Scale

#### 2.1.1. General Considerations about Smartphone Acoustic Calibration

The principle of involving citizens in a participative science approach in the acoustical context is to collect massively geo-localized objective and subjective acoustic data. These data can then be used to produce noise maps for the benefit of local authorities, for example, in the context of establishing action plans to reduce noise pollution. The project can also be part of an educational [13,14] or citizen approach to raising awareness and the co-construction of public policies [15,16]. Whatever the purpose of the collected data, the calibration of smartphones is an issue that is often discussed.

Several works have shown that different acoustic measurement applications installed on the same smartphone or the same application installed on different smartphones can generate differences in the measured acoustic indicators [17,18,19] that can reach up to nearly 30 dB compared to a reference device [20]. It can be explained in particular by the different coding of the applications as well as by hardware differences between the smartphones. In this context, particular attention was paid to the development of the NoiseCapture application to ensure compliance with the acoustic acquisition protocol on Android smartphones. One can expect that the dispersion of measured noise values within the NoiseCapture application is lower. However, the calibration of the application/smartphone pairs is still required to obtain acoustic results with a minimum of bias [18,20,21].

In this paper, acoustic calibration is seen as the correction of a measured sound pressure signal so that this measurement coincides with a reference signal (i.e., an acoustic calibrator most of the time). This correction allows for a systematic error between the device to be calibrated and the reference device. In the simplest case, if *X* is the temporal sound pressure signal measured by the smartphone, then the true value *Y* of the observable is related to the measured measurement *X* via a calibration coefficient *k* such that:

(1) Y=k×X.

Within a smartphone application for noise measurement, the calibration consists of estimating this coefficient *k*, which normally takes into account all the elements of the analog–digital conversion chain, such as the correction linked to the sensitivity of the microphone and the effects of the digital discretization of the signal. Considering sound level in decibels (dB) instead of acoustic pressure, the estimated sound level $L_{Y}$ can be calculated using the measured sound level $L_{X}$ by the smartphone with the following relation:

(2) $$L_{Y}=L_{X}+20\log k=L_{X}+\Delta .$$

Without the correction, the smartphone will produce a systematic offset (in dB) of a value equal to Δ.

In most experiments, the calibration procedure consists of evaluating the difference Δ in measurement between a smartphone and a reference device (e.g., a class 1 sound level meter) and then proceeding to a correction in the overall sound level, possibly A-weighted, by using an acoustic correction factor [22]. Most of the time, this correction is assumed to be a constant compared to the reference device; however, linearity problems can occur at low and high levels and in frequencies, which could justify a more adapted calibration [20,21], such as proposed by [23] for example. Instead of using reference devices, some alternative calibration methods have also been proposed, based, for example, on the measurement of a quiet sound level [18] or on the in situ measurement of road traffic noise [24]. In addition, if the calibration corrections are collected for different smartphone models and integrated in a reference database, the calibration of a smartphone can also be performed indirectly by searching for the corresponding calibration value in this database [18]. Nevertheless, some works have also shown possible differences between two identical models of smartphones, depending on different versions of the operating system or due to hardware changes on two generations of the same model [20]. Note also that the use of an external microphone instead of the smartphone’s internal microphone can improve the accuracy of the measurement, but it still requires microphone calibration [25,26,27,28].

#### 2.1.2. Smartphone Calibration with NoiseCapture

Like other similar applications, NoiseCapture allows for defining a calibration value Δ either directly by manually entering a calibration value in the application parameters or automatically using one of the calibration methods proposed by the application: using either an acoustic calibrator, a reference measurement device, a smartphone already calibrated with the NoiseCapture application, or based on a road traffic measurement. This calibration can be carried out several times, possibly giving rise to different calibration values, but for each measurement carried out, the calibration value applied is systematically associated with the measurement track (i.e., for all the measurement points in the corresponding track). In the first public release of the application, only the calibration value was collected. Since NoiseCapture release 51 (1.2.15) at the end of 2020, the method used for calibration is also part of the information collected.

In practice, over the period 2017–2020 (before release 1.2.15), 24% of measurement points have a calibration value different from the default value (0 dB), which suggests that the corresponding smartphones may have undergone acoustic calibration. However, even though it represents a very large mass of data, the observed calibration values may call into question the quality of the calibration: 61.12% of the calibrated smartphones, for example, have calibration values higher than ±15 dB, which does not seem realistic even considering the low metrological quality of some smartphones. Finally, only specific events organized by specialists, for example with the objective of raising awareness among citizens or for research purposes, can ensure a high quality of data by considering a state-of-the-art calibration and a training of the users [22,29,30]. It is particularly the case for NoiseCapture Party events, which aim at collecting data during a specific event, supervised by qualified persons, generally over a short period of time and a limited spatial extent. However, such data represent only 0.6% of the data collected over the 2017–2020 period [9].

#### 2.1.3. Mass Calibration vs. Individual Calibration of Smartphones

Relevant exploitation of mobile data at a large scale is therefore hampered by the heterogeneity of the collected data, which is mainly due to the lack or misapplication of a calibration protocol. To solve this problem, a relevant solution consists of simultaneously calibrating a posteriori all the collected data, including those that would have given rise to a calibration, in order to ensure total coherence between the data. In the literature, this mass calibration of data measured with mobile sensors, instead of considering the individual calibration of sensors, has led to the development of specific methodologies referred to as blind calibration, self-calibration, or re-calibration. In [31], the authors propose, for example, to take advantage of the multiple rendezvous between an uncalibrated smartphone and several calibrated smartphones to estimate its bias; a consensus is then found to calibrate all the smartphones simultaneously by solving a discrete average consensus problem. Here again, the fact of having only a few reference data points limits the use of the method. On the contrary, in [32,33], the Moments-Based Calibration approach does not require reference data but considers that all mobile sensors move in the same way in the whole study domain with the same probability. The ergodicity property then simplifies the mathematical analysis of the problem; in practice, as in our case, it is however not verified, since at the scale of a large territory, it is admitted that two smartphones will never meet. In [34], the calibration method does not rely on any such assumption and formulates the mutual calibration problem as a linear algebra problem whose solution relies on the resolution of a Laplacian matrix. This last method seems particularly well-suited to NoiseCapture data, and we have therefore chosen to adapt it to the present problem.

### 2.2. NoiseCapture Application and Database

#### 2.2.1. Application Principle

**Figure 1:**
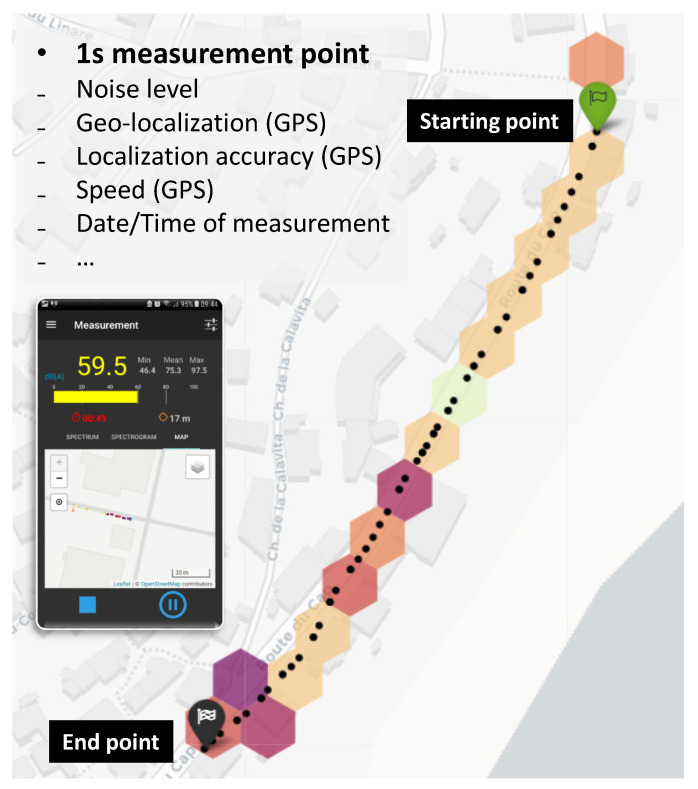
NoiseCapture approach. Using the NoiseCapture application, a user moves along a path (i.e., a track in the NoiseCapture vocabulary); each second (i.e., a measurement point), several noise indicators (sound level, spectrum) and other information (date/time, localization, speed, etc.) are calculated. When the user stops the measurements, the data are stored within the smartphone, and, if authorized by the user, uploaded to the NoiseCapture remote server. Raw data collected by the entire NoiseCapture community is preprocessed and averaged in hexagonal spatial zones (15 m radius); then, they are displayed in the form of noise maps.

The principle of mobile noise measurements is to collect geo-referenced acoustic data in a spatial area (Figure 1). A given user starts a measurement, moves along a path, and then stops the measurement. At each time step of 1 s, several acoustic indicators are calculated on the fly, recorded on the smartphone, and sent anonymously to a remote server. The transmitted data are verified and archived, and then they are processed in a simplified way in order to represent them in a cartographic representation. This representation takes the form of a noise map, where some acoustic indicators are aggregated on a hexagonal elementary spatial extent, the network of hexagons covering the entire globe.

#### 2.2.2. Database and Privacy Policy

All data collected by the application are detailed in reference [35]. Particular attention has been paid to strict respect for privacy as well as the use of these data by the contributor and by third parties. The application’s data confidentiality policy clearly explains the purpose of the application, namely to meet the needs of scientific research, in a context of participatory science and open science, carried out by French public research establishments (Université Gustave Eiffel—formerly Ifsttar—and CNRS). It is also specified that no personal data are collected. The development of this application and the redaction of the privacy notice was performed in consultation with the departments in charge of open science and legal aspects at Université Gustave Eiffel—formerly Ifsttar—in 2016–2017.

In particular, it is clearly specified from the very first run of the application that its use does not require registration, does not collect any personal data, does not record any audio data (acoustic indicators are calculated on the fly), and does not perform any background tasks. Furthermore, it is specified that the user can choose whether or not to contribute to the community database, can stop data collection at any time (by no longer using the application or by uninstalling it from the smartphone), and can access the application’s source code and all data collected by the community. This short version of the privacy policy refers to a detailed online version on the NoiseCapture application website.

Each installation of the smartphone application creates a unique universal identifier (UUID), which is then associated with each set of measurements carried out with the smartphone. This enables all the data collected by a single smartphone to be postprocessed under a relevant process (for example, in this case, for the blind calibration of the smartphone measurements). This UUID is not linked to any other smartphone identifier. In addition, a user can generate a new UUID by uninstalling and reinstalling the application. If a user contacts the NoiseCapture project administrators to have their measurement data removed from the community server, they will be asked to provide this UUID.

From an application functional point of view, and like any application developed on the Android platform and deposited on the official repository (Google Play), the person installing and using the application must validate authorizations. In our case, only two authorizations are required: device location authorization and audio recording authorization. When the application is run for the first time, the short version of the NoiseCapture privacy policy is displayed and detailed [36], and the user must validate and accept these conditions in order to use the application. Also on first execution, a text is displayed to justify the application’s activation of device localization (NoiseCapture localization policy [37]). Users are also regularly warned that this application can never replace a calibrated professional sound-level meter.

The data collected by the application and uploaded to the community server are accessible in their entirety, free of charge, and distributed under the Open Data Commons Open Database License (ODbL) [38], meaning that it is possible to share (to copy, distribute and use the database), to create (to produce works from the database) and to adapt (to modify, transform and build upon the database) as long as the database user attributes, shares and keeps open the database. This seemed to us to be a fair return for the community contributing to the development of this database and also in the context of Open Science.

### 2.3. Blind Calibration Model

#### 2.3.1. Natural Graph Model

**Figure 2:**
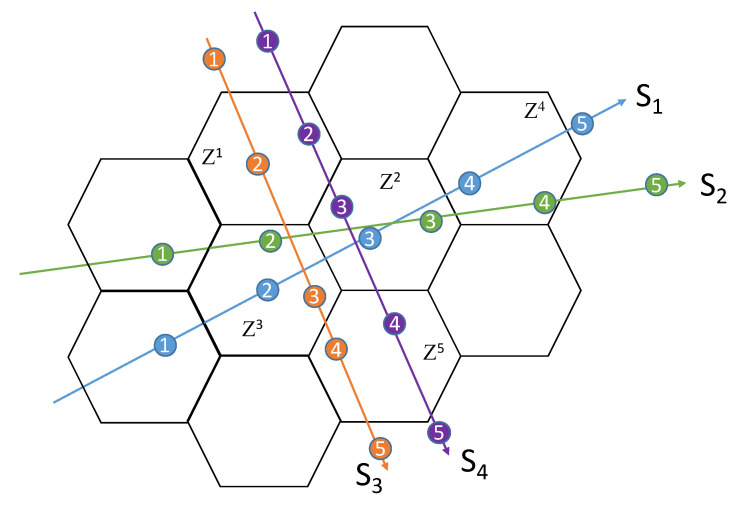
Principle of the blind calibration methodology applied to mobile noise measurements. During the procedure, several sensors noted that $S_{1}$, $S_{2}$, $S_{3}$, and $S_{4}$ crossed the same spatial area (i.e., an hexagonal zone, noted $Z^{i}$) at the same time ($t_{1}$, $t_{2}$, $t_{3}$, $t_{4}$, $t_{5}$). In theory, these sensors should measure exactly the same acoustic event and therefore produce the same noise indicators. The path of a user is symbolized by a colored arrow; at each time step, the user is localized at a given position, which is symbolized by the colored circle with the time increment inside.

Among the solutions proposed in the literature for blind calibration, as a first attempt, the Natural Graph Model (NGM)-based blind calibration scheme proposed in [34] seems adapted to the mobile noise measurements, such as those collected using the NoiseCapture application. This method consists of exploiting the multiple appointments of sensors at positions close in time and space (i.e., in the same hexagon at a nearby time period) in order to establish mutual calibrations between sensors (Figure 2). In other words, if two smartphones simultaneously measure the same acoustic phenomenon, they should produce the same indicators (in the next development, we will say that there is a link between the two smartphones). This approach assumes that the sound level is homogeneous in a hexagonal zone whatever the position of the smartphones in that zone. There is no interaction between the zones; they are independent.

Due to differences in calibration for both smartphones, this rendezvous leads to the establishment of a correction factor between the two smartphones, i.e., a relative calibration, which can be generalized to the scale of a network of smartphones to establish relative calibrations between devices. For a very dense sensor network, the multiple appointments create a redundancy of information, which can also be exploited to improve the quality of the calibration.

In the following, the original NGM methodology [34] is detailed and applied to the mobile noise measurement, using the same notations. However, we do not repeat all of the original developments so as not to make this article too long. Readers are invited to consult the original article.

Let us consider, for example, four sensors ($S_{1}$, $S_{2}$, $S_{3}$, $S_{4}$) traveling a path passing indifferently several hexagons covering a spatial extent at different times *t*. All the users numbered *i* present at the same time *t* in the same area define a zone *Z* of sensors that measure the value *x* of the same observable *y* of the event. Using the relation (2), we have [34]:

(3) $$y=x_{i}+\Delta_{i}=x_{i}+d_{i}+n_{i}.$$

In the relation, it is assumed that the offset Δ that is estimated for a sensor is the sum of the exact drift *d* related to the calibration, assumed to be systematic and stationary over time, and an error *n* is associated with a non-predictable external effect and non-systematic, which is assumed to be white noise.

Thus, we can define the zone $Z^{\alpha }$ containing $N^{\alpha }$ co-located sensors (Table 1), performing the measurement $x^{\alpha }$ of the same observable $y^{\alpha }$ (i.e., the true value) such that [34]:

(4) $${\left\{y^{\alpha }=x_{i}^{\alpha }+d_{i}+n_{i}^{\alpha }\right\}}_{S_{i}\in Z^{\alpha }}.$$

For each sensor $S_{i}\in Z^{\alpha }$, the corresponding drift $d_{i}$ can thus be expressed by the other smartphone drifts $d_{j}$ ($S_{j}\in Z^{\alpha }$, $S_{j}\neq S_{i}$) using the following relation [34]:

(5) $$d_{i}=\frac{1}{N^{\alpha }-1}\sum_{\left(S_{j}\in Z^{\alpha },S_{j}\neq S_{i}\right)}\left(d_{j}+\Delta x_{ji}^{\alpha }+\Delta n_{ji}^{\alpha }\right),$$

with $\Delta x_{ji}^{\alpha }=x_{j}^{\alpha }-x_{i}^{\alpha }$ and $\Delta n_{ji}^{\alpha }=n_{j}^{\alpha }-n_{i}^{\alpha }$.

Since the sensor *i* moves along other zones and the drift $d_{i}$ is stationary over time, one can derive a set of linear equations. Considering the whole set of sensors, the linear equations can be written following a matrix form [34]:

(6) $$L\vec{d}=\Delta \vec{x}+\Delta \vec{n},$$

where L is the calibration matrix, $\vec{d}$ is the drift vector, $\Delta \vec{x}$ is the differential vector, and $\Delta \vec{n}$ is the differential white noise vector. Due to the properties of L, the calibration matrix is the Laplacian matrix. Lastly, the authors consider two more hypotheses: (1) the differential white noise vector $\Delta \vec{n}$ is negligible when considering a large number of sensors, meaning that $L\vec{d}\approx \Delta \vec{x}$; (2) the mean value of all smartphone drifts is nearly zero, which leads to the equivalent constraint $M_{1}\vec{d}=0$ where the elements of $M_{1}$ are all equal to 1. Finally, the authors show that the drift vector can be obtained by resolving the following matrix inversion [34]:

(7) $$\vec{d}={\left(L+M_{1}\right)}^{-1}\Delta \vec{x}.$$

Once the drift vector is obtained, the estimated true value in a zone can be calculated using relation (4).

#### 2.3.2. Simple Mean Model

Instead of using the NGM methodology, one can consider a very simplified approach, the Simple Mean Model [39], which is also considered in the reference works for predicting the gain calibration value for each smartphone. First, we take the average of the measurement values in each column of Table 1 to estimate the true input value of a zone. The SMM assumes a large number of sensors and estimates the true input value $y^{\alpha }$ using:

(8) $${\hat{y}}^{\alpha }=\frac{1}{N^{\alpha }}\sum_{\left(S_{i}\in Z^{\alpha }\right)}\left(x_{i}^{\alpha }\right)=\frac{1}{N^{\alpha }}\sum_{\left(S_{i}\in Z^{\alpha }\right)}\left(y^{\alpha }-d_{i}-n_{i}^{\alpha }\right).$$

Next, the drift value of a sensor can be estimated by calculating:

(9) $$d_{i}={\hat{y}}^{\alpha }-x_{i}^{\alpha }.$$

The linear Equation (5) for the NGM model, plus the constraint $\sum_{i}\left(d_{i}+n_{i}^{\alpha }\right)\approx 0$, is then equivalent to the SMM. In other words, the NGM is a generalized extension of the SMM.

#### 2.3.3. Validation of the NGM Implementation

The NGM implementation was validated by direct comparison with the results published in the reference article [34] for a test dataset. This dataset is based on S=100 simulated measurements located in G=100 zones. Each measurement is simulated as the sum of the true value of the measurement *y* (a random number between 0 and 100 according to a uniform distribution), of a drift *d* (a random number according to a Gaussian distribution of variance $\Delta_{drift}$) and of a noise *n* (a random number according to a Gaussian distribution of variance $\Delta_{noise}$). The membership of a measurement in a zone is obtained randomly. Note that at this step, this dataset has no relation to sound levels and is only used for evaluating the NGM behavior.

**Figure 3:**
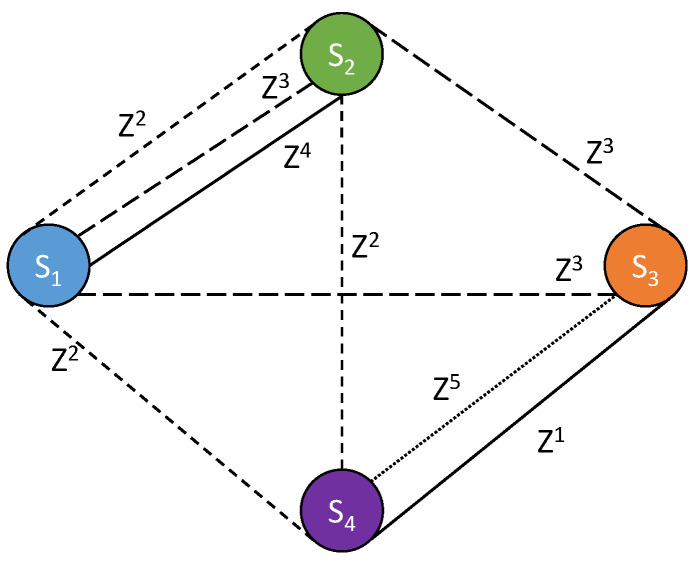
Network graph based on the scenario of Figure 2 and Table 1.

**Figure 4:**
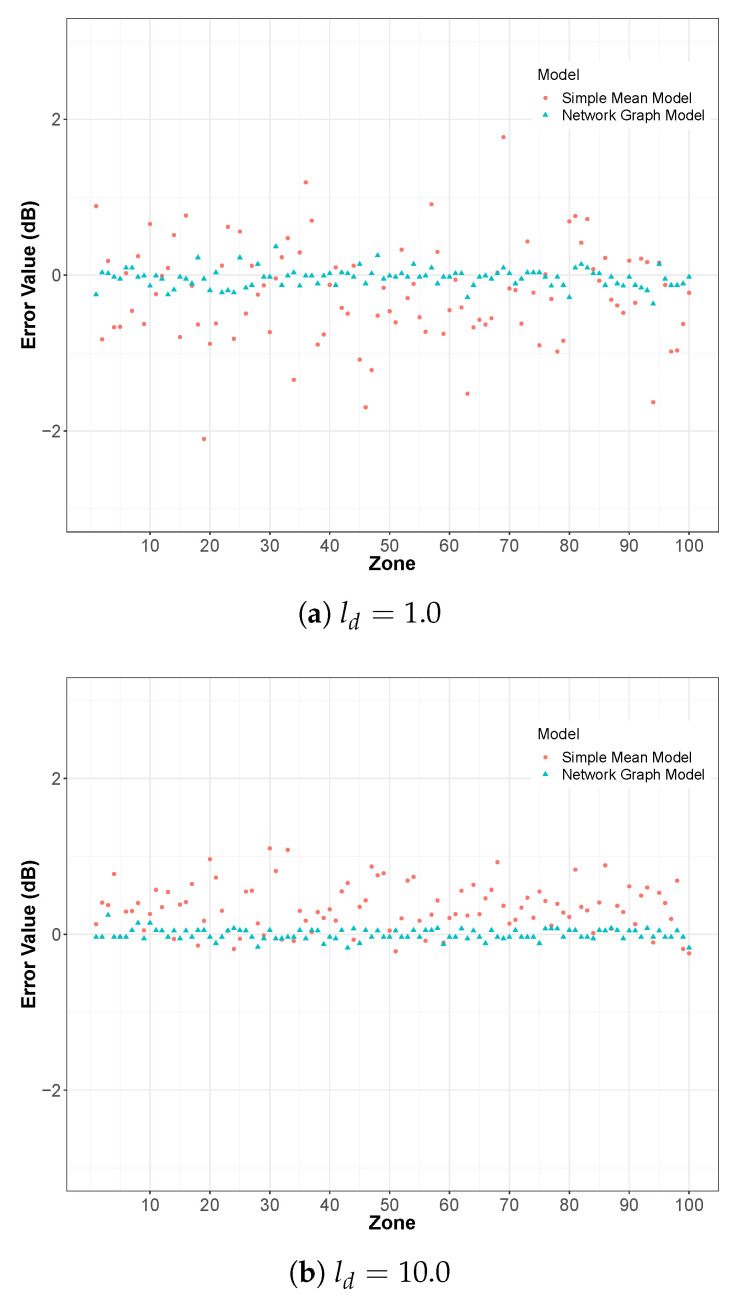
Comparison between the NGM and the SMM: error between the true value and the estimated value, for a link density (**a**) $l_{d}=1.0$ and (**b**) $l_{d}=10.0$, with N=100 smartphones in *G* zones.

**Figure 5:**
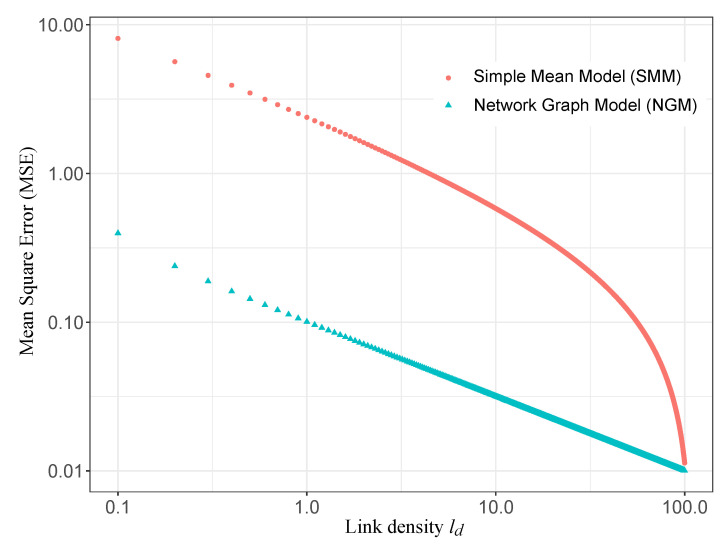
Comparison between the NGM and the SMM: mean square error in function of the link density $l_{d}$, with N=100 sensors in *G* zones.

On the basis of this dataset, a network graph can be generated to (Figure 3). The system (6) is then solved in order to determine the estimated value of the drift according to the relation (7) as well as the estimated value of the measurement in the corresponding zone according to the relation (4). The Mean Square Error (MSE) between the true value *y* and the estimated value $\hat{y}$ can then be computed in order to evaluate the model efficiency. In the reference article, the authors choose to represent the results through the link density metric $l_{d}\equiv 2L/\left[S(S-1)\right]$, which represents, on average, the number of times a given smartphone encounters other smartphones, with *L* designating the number of links. In addition to the application of the present NGM, the results obtained by the Simple Mean Model defined in Section 2.3.2 are also represented. The results are presented in the two following Figure 4 and Figure 5, and they are very similar to Figures 3 and 4 of the reference article [34]. This simple comparison validates our implementation of the NGM.

Figure 4 illustrates the error between the estimated value and the true value of the measurement for two values of the link density ($l_{d}=1.0$ and $l_{d}=10.0$). As expected, when the number of links between sensors increases (when $l_{d}$ increases), the estimation error decreases. Moreover, this figure shows very clearly that the NGM gives a better estimation than the SMM.

Figure 5 generalizes this conclusion by summarizing the results for several values of the link density $l_{d}$. The NGM converges quickly to the true values, even for low link densities, while the SMM requires a larger number of links to reach an equivalent level of performance.

From a practical point of view, the optimization of the results of the model requires both an increase in the number of sensors and in the link density. Understandably, the more links there are between different sensors and the higher the number of sensors, the better the results.

## 3. Application of the NGM to a Mobile Acoustic Dataset

### 3.1. Discussion of NGM Application Assumptions

The development of the NGM is based on several assumptions that need to be discussed regarding its applicability to a mobile acoustic data dataset. Overall, the reliability of all these assumptions, although questionable, is also supported by the results that will be presented later.

#### 3.1.1. NGM Mathematical Assumptions

Regarding the mathematical assumptions of the model, one can consider the following discussion:

- First assumption: the drift *d* of a given sensor is stationary over time. In principle, the variation of drift over time of a professional microphone is small, especially with respect to its impact on measured noise indicators. A smartphone microphone, on the other hand, is exposed to numerous constraints that may partially modify its acoustic characteristics over time. To our knowledge, there is no published study on the acoustic monitoring of smartphones over time, at least for environmental acoustics applications, but our experience within the NoiseCapture project has not revealed any anomalies on this subject. Moreover, considering the rapid change in the smartphone fleet, the assumption of stationarity over a short or medium time period seems quite acceptable. In the event of a full deterioration of the smartphone microphone, following an accident, for example, the smartphone will become unusable for its primary function, and it is likely that it will no longer be used to collect data.
- Second assumption: the average value of drifts *d* on all sensors is null. The average value of all known calibration values in the NoiseCapture database, if we exclude calibration values at zero (default value in the absence of calibration), is of the order of −0.43 dB, i.e., close to zero. This hypothesis, therefore, seems globally acceptable. It is important to note first of all that this assumption is introduced by the authors to ensure the uniqueness condition of the solution of Equation (6) [34]. The assumption can therefore be discussed but is, in any case, required in the approach.
- Third assumption: the noise vector $\vec{n}$ is small in front of $\vec{x}$ for a large number of sensors. It is difficult to quantify the error introduced by external conditions or insufficient control of the measurement protocol (noise generated by the operator, bad holding of the smartphone, effect of the wind on the microphone, etc.). However, one can consider that this noise is negligible in comparison with the measurement, and that it can be assimilated to a white noise.

#### 3.1.2. Sensor Definition in the Context of a Mobile Acoustic Measurement

It is also important to consider the definition of a sensor in the context of a mobile acoustic measurement. Indeed, in the present application, we consider a sensor as a (smartphone model, NoiseCapture user) pair (noted later as a (smartphone, user) pair), even if several users can use the same smartphone model. It allows us to consider a specific calibration for each pair: it enables taking into account the fact that two users can, for example, use the same smartphone model with a different measurement protocol, or that the same smartphone model can give rise to several technically different generations and then different calibration corrections. In the NoiseCapture approach, a given user is defined by an Universally Unique Identifier (UUID) that is associated to the corresponding smartphone.

#### 3.1.3. Assumption of Simultaneous Measurements between Two Sensors

The major assumption of the NGM model, which requires matching data that were measured at the same time and at the same place, is very crucial and raises the question of the choice of “homogeneous” time periods for the collected data in the context of a mobile acoustic dataset. In reference [40], the authors consider, for example, that a measurement of 10 min duration can be sufficient to characterize the sound environment equivalent to a period of one hour and that “homogeneous” periods of the same day can be discriminated by measurements of 10 to 20 min. For the moment, the temporal distribution of the collected data with NoiseCapture is not controllable, and only the accumulation of a large number of data with time will be able to ensure, in the future, a sufficient number of data for all temporal and homogeneous reference periods of a day. At this stage, within the framework of the present work, we will consider a larger time period of 1 h or more with the hypothesis of homogeneous sound environments.

### 3.2. Comparison with Reference Datasets: NoiseCapture Parties

In the NoiseCapture approach, specific events can be specifically organized in order to collect acoustic data over a defined spatial extent and over a given period. These events, called NoiseCapture Parties, are organized, for example, by researchers to collect data on a specific territory as part of their research into exposure to noise pollution [41,42,43], by teachers to train school and university students in environmental noise issues [14], or by local authorities wishing to raise awareness on the subject of noise environments [16]. In general, these events are run by professionals who are very familiar with the practice of acoustic measurement in the environment. For such events, smartphone calibration is systematically provided and the measurement protocol is detailed. Therefore, on these reference datasets, some calibration data are available for a large number of smartphones (i.e., the initial calibration value).

In this section, and as a preliminary step, we propose to apply the NGM to several reference datasets (Table 2). Each dataset is defined by an identifier ‘pk_party’ that identifies the corresponding data in the reference database [9]. The total number of 1 s measurement points, the number of tracks (consisting of all 1 s measurement points during the same track), the measurement time period, as well as the total number of (calibrated) smartphones, are also indicated. In addition, in the framework of the application of the NGM model to these datasets, the number of links and the value of the link density $l_{d}$ are also given.

Each event allows for the collection of data on a spatial extent defined by a set of contiguous hexagonal areas, as illustrated for example in Figure 1. The rayon of the hexagons is set to 15 m by default in the NoiseCapture approach, but the influence of this size on the behavior of the model will be discussed later in Section 3.4.

**Figure 6:**
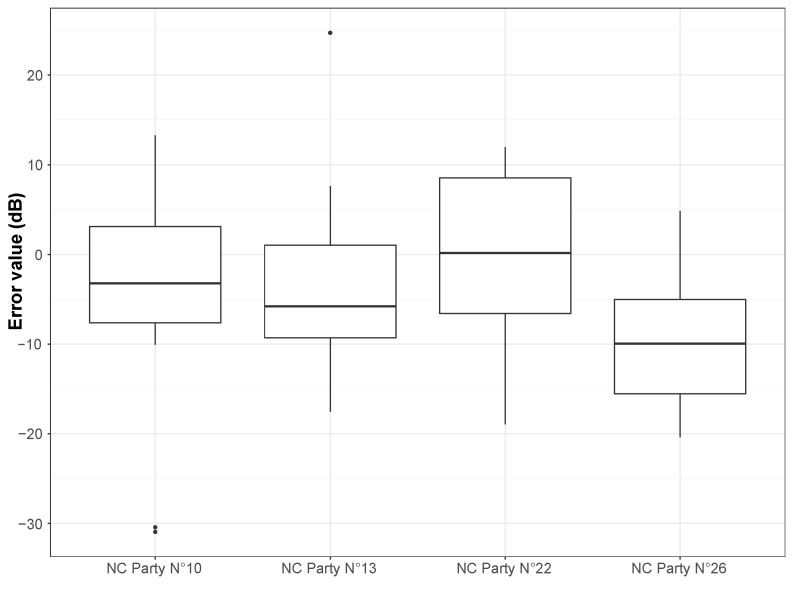
Error value between the estimated drift value and the initial calibration value for each calibrated smartphone used in the NoiseCapture (NC) Parties N°10, 13, 22 and 26.

By construction, it is expected that the NGM performance will increase as the number of links between sensors increases and, therefore, as link density increases, too. In view of the $l_{d}$ values in Table 2 and by looking at Figure 6, this hypothesis does not appear so clearly, even if the trend is globally respected.

**Figure 7:**
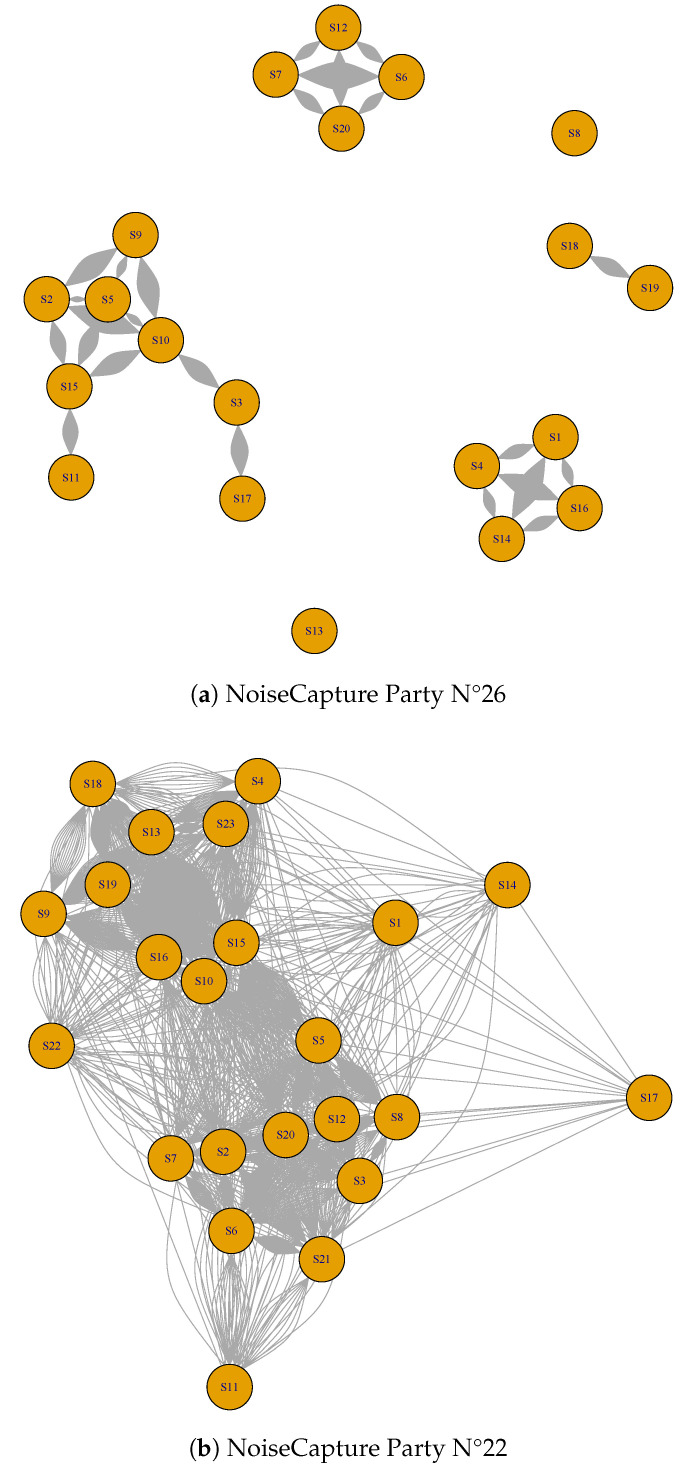
Smartphone network graph for the NoiseCapture Party (**a**) N°26 with 20 linked smartphones within 6 distinct subsets of data and (**b**) N°22 with 23 linked smartphones within the same subset of data.

Beyond a high $l_{d}$ value, it is important that all smartphones are linked together. For example, in the case of NoiseCapture Parties N°13 and 26, one can observe that there are several groups of smartphones, with many links within each of these groups but not between smartphones from different groups (see, for example, Figure 7a for the NoiseCapture Party N°26). Conversely, the NoiseCapture Party event N°22 yields satisfactory results because most of the sensors are linked together (see Figure 7b for the NoiseCapture Party N°22). However, the variance is greater for NoiseCapture Party N°22, but this can be explained by a longer measurement period (7 h) than for the other NoiseCapture Parties, possibly generating a greater variability in sound levels.

### 3.3. Hybrid NGM-SMM

As discussed in the last paragraph, the improvement of the NGM method relies on the increase in the number of links between smartphones and, thus, the increase in link density. Obviously, if there are too many smartphones with few links with other smartphones, then the link density will decrease and the model efficiency will also decrease. An alternative to the original approach consists of applying the NGM to the pairs (smartphone and user) with the most links and then using the corresponding calibrated pairs to determine the drift of the other pairs by using SMM. This methodology, which can be qualified as a hybrid NGM-SMM method, makes it possible to “focus” the NGM efficiency on the most relevant pairs by optimizing the link density and to determine the calibration values for the other pairs more easily with the SMM.

This methodology has been first tested on the dataset of the NoiseCapture Party N°22, but the conclusion is similar for the other NoiseCapture Parties. Several values of the minimal number of links per pair (smartphone, user) to be considered as a cut-off between NGM and SMM in the hybrid method were tested: from more than 1 link (this corresponds to the full NGM, with 23 (smartphone, user) pairs) to more than 140 links (12 remaining pairs), in order to evaluate the hybrid model efficiency. As expected, when the minimum number of links increases, the number of remaining (smartphone, user) pairs naturally decreases.

**Figure 8:**
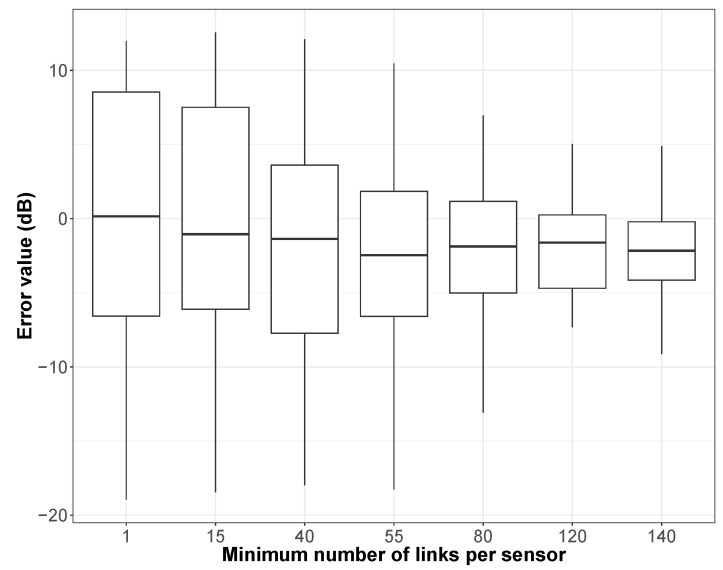
Application of hybrid NGM-SMM methodology on the NoiseCapture Party N°22 dataset. Error (in dB) between the estimated drift and the initial calibration of smartphones as a function of the number of links between (smartphone, user) pairs from 1 (this corresponds to the full NGM, i.e., the reference using the initial 23 smartphones) to 140 (12 remaining smartphones).

Figure 8 illustrates the results of this hybrid method through the mean error between the estimated drift values and the initial smartphone calibration values. In these results, all smartphones are concerned, whether they have been calibrated by the NGM method or by the SMM method. Compared to the NGM reference, we observe a better behavior of the hybrid approach (the variance decreases), and this is more so as the minimum number of links increases. This result clearly shows the contribution of the hybrid NGM-SMM method compared to the NGM method alone.

### 3.4. Effect of the Size of the Spatial Area on the Hybrid Method

As mentioned below, the size of the spatial area may have an effect on the method’s efficiency. In this paragraph, we compare the effect of the size of the hexagon on the result of the hybrid model using the NoiseCapture Party N°22 dataset. Results are detailed in Table 3 in terms of mean error (in dB) between the estimated drift and the initial calibration value and in terms of uncertainty (i.e., the interval between the 75 and 25 quantiles after correcting with the bias value). It should be noted that the larger the area, the fewer the links between smartphones; this explains why some of the rows in the Table 3 do not give any results. Whether for the mean error or for the uncertainties, the results in Table 3 show that for the corresponding dataset, the best compromise is obtained for a hexagonal size of 15 m. These results confirm the initial hypothesis of the NoiseCapture approach, suggesting that the sound environment may be considered as homogeneous in an area of 15 m size.

### 3.5. Comparison with Large Realistic Dataset: City of Rezé (France)

#### 3.5.1. Description of the Dataset

**Figure 9:**
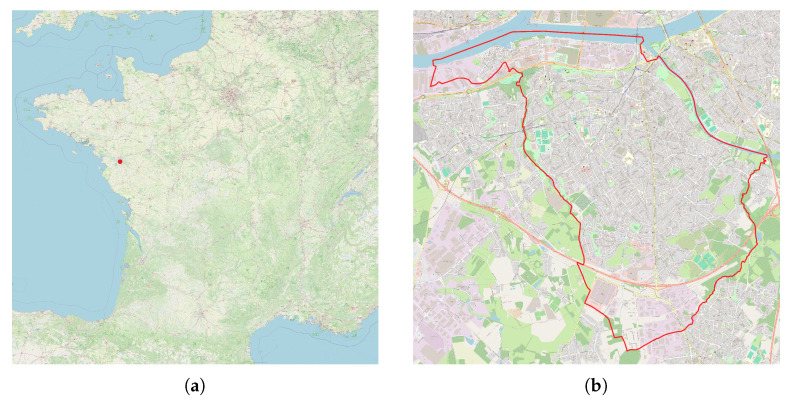
Localization of the City of Rezé in France. (**a**) Localization of the City of Rezé (France); (**b**) Boundaries of the City of Rezé (France).

**Figure 10:**
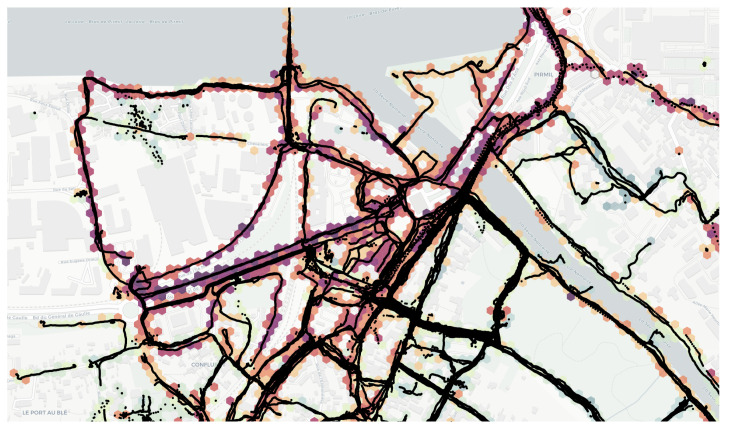
NoiseCapture data collected on a small part of the City of Rezé in France: measurement points and noise map (in dBA) built with raw data.

The previous analysis is now extended to the City of Rezé, part of the Nantes metropolitan area, in France (Figure 9), for which a very large amount of data has been collected, both in the context of NoiseCapture Party events (NoiseCapture Party N°2, N°9, and N°52) and by “independent” contributors. In this area, additional data have also been collected similarly to a NoiseCapture event, in the framework of the Sonorezé research project [16], but are not a part of NoiseCapture Parties. The involved area represents a surface of 13,780,000 m^2^, gathering a total of 450,335 of 1 s measurement points and 2336 tracks on 10,365 hexagons (Figure 10), collected by 331 (smartphone, user) pairs with 163 different smartphone models. Reference data (NoiseCapture Parties) represents 1877 of 1 s measurement points (0.4% of the whole dataset) and 16 tracks (0.7%), collected by 4 (smartphone, user) pairs (1.2%) and 3 different smartphone models (1.8%). Of the 331 pairs, only 134 smartphones were calibrated by users, which corresponds to 278,561 (61.9%) of 1 s calibrated measurement points and 1529 (65.5%) calibrated tracks. The map shown in Figure 10 is obtained by averaging the sound levels at all the measurement points in each hexagon over the entire data collection period [35].

This dataset was collected over 6 years (2017–2023) at different times of the day and on different weekdays and weekends. In the present work, we have chosen to limit the application of the hybrid method to 08:00–20:00 (as a unique time period), for which a large number of datasets are available, considering that the long-term sound environment would be homogeneous during these periods. It corresponds to 315,598 of 1 s measurement points (i.e., 70.1% of the initial dataset) and 1712 tracks (73.3%). Moreover, to avoid the high variation when it comes to short measurements, a more ‘homogeneous’ approach was considered. This approach was to consider (smartphone/user) measures that stay less than 30 s (it corresponds to 65.3% of the initial dataset in terms of measurement points), 20 s (71.6% of the initial dataset) or 10 s (91.8% of the initial dataset) in each hexagon. These sub-datasets will be referred to in the next paragraph to as ‘filtered data’ (Table 4).

#### 3.5.2. Time Slot Variability for a Rendezvous

**Figure 11:**
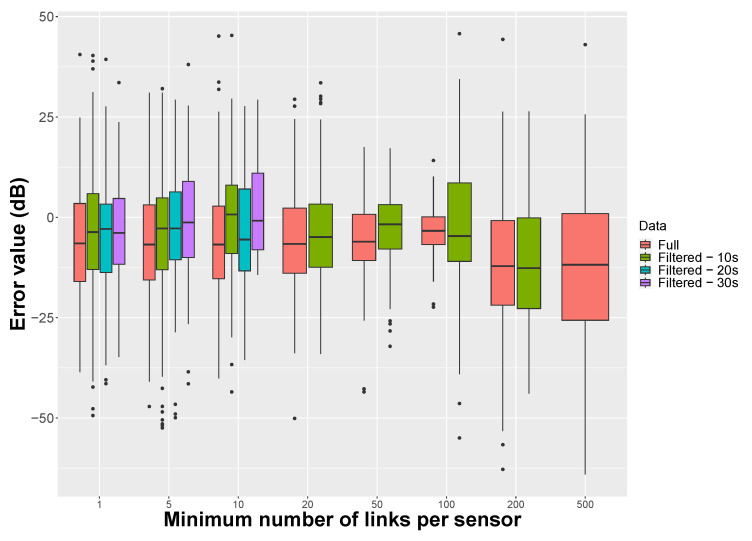
Application of the hybrid NGM-SMM methodology on the City of Rezé. Mean error (in dB) between the estimated drift and the initial calibration of smartphones as a function of the number of links between couples (smartphone, user) from 1 (i.e., the NGM reference) to 500, for each filter duration. The hybrid method is applied to both the ‘full’ dataset and the sub-dataset (‘filtered’) that correspond to a presence time of at least 30 s, 20 s and 10 s in a hexagon area.

Similarly to Figure 8, Figure 11 illustrates the mean error and uncertainty of the hybrid method, applied to data collected for the City of Rezé, as a function of the minimum number of links between (smartphone, user) pairs. Overall, we can already see that the variance is greater with this Rezé dataset than for the results shown in Figure 8 for the NoiseCapture Party N°22. It is due to the fact that this dataset contains a large amount of data produced outside the NoiseCapture Parties, some of which is of lower quality.

The approach is also applied on the sub-dataset with a minimum presence time of 30 s, 20 s and 10 s in a hexagon. Here again, the hybrid approach seems to give better results as the number of minimum links increases (the mean error decreases, as does the uncertainties). For the full dataset, the limit of improvement is reached a priori when the number of remaining (smartphone, user) pairs becomes insufficient. In the present case, this limit seems to appear for a number of links between 50 (94 remaining pairs) and 200 (37 remaining pairs), and it is visible for a number of links equal to 100. In this case, the average error is −3.4 dB between the smartphone calibration values and the drift values obtained using the hybrid method. The uncertainty is also much lower in this situation.

When considering a minimum time of presence in an hexagon area, we observe that the mean error decreases in comparison with the full data (results for a minimum number of links of 5 and 10), while the uncertainty is quite similar and constant. For a larger number of links, there are no more remaining (smartphone, user) pairs, and the hybrid method cannot give a result. When comparing the results for the full dataset with the results for a time of presence of 10 s, we observe that the optimum minimum number of links is reached earlier for the filtered data. It is difficult to conclude, since there are not enough data for 20 and 30 s, but one could expect that increasing the temporal filter duration will increase the quality of the results of the hybrid method.

#### 3.5.3. Qualitative Results

**Figure 12:**
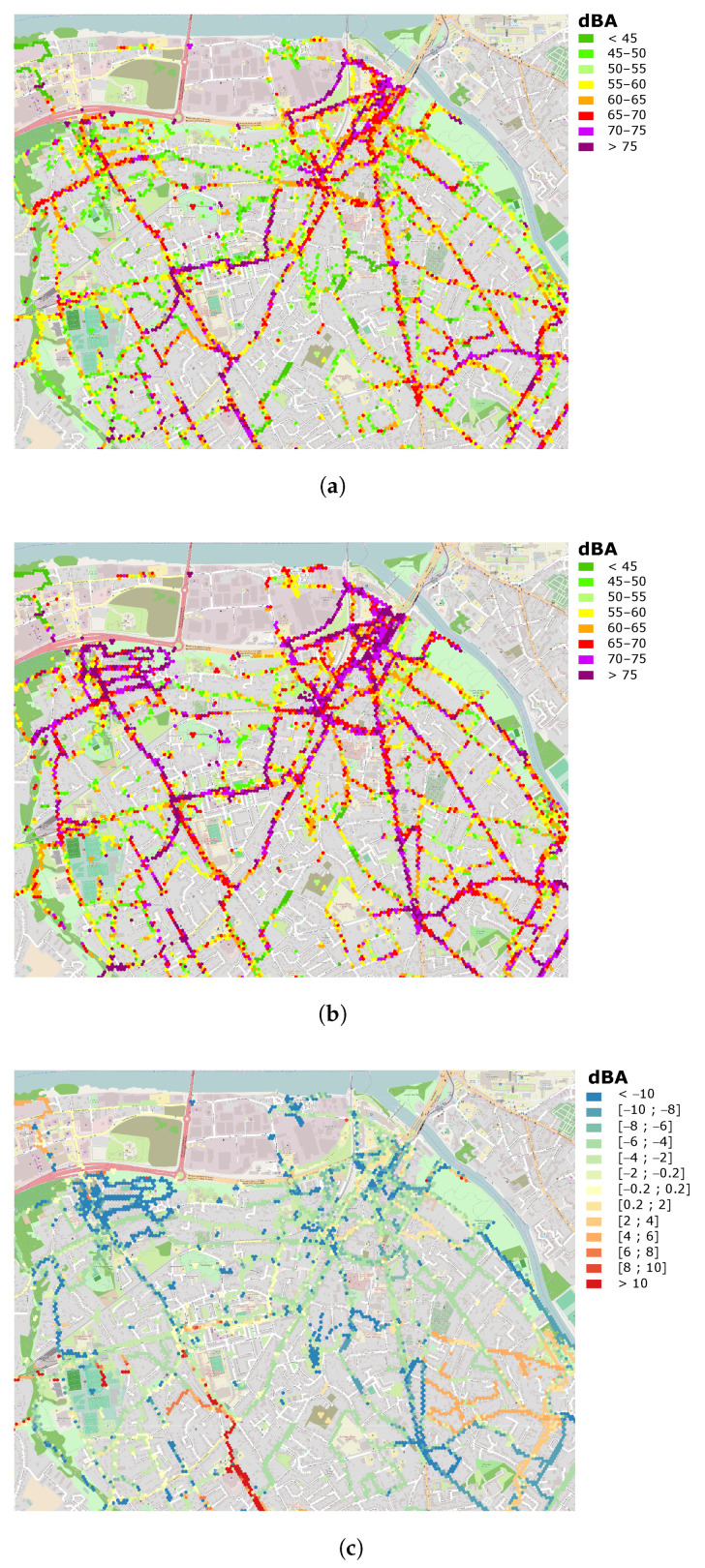
Noise maps of a part of the City of Rezé: (**a**) data with initial calibration (134 calibrated (smartphone, user) pairs); (**b**) data after applying the blind calibration on the initially calibrated smartphones only; (**c**) difference noise map (**a**,**b**), see also details of the differences in Figure 13.

**Figure 13:**
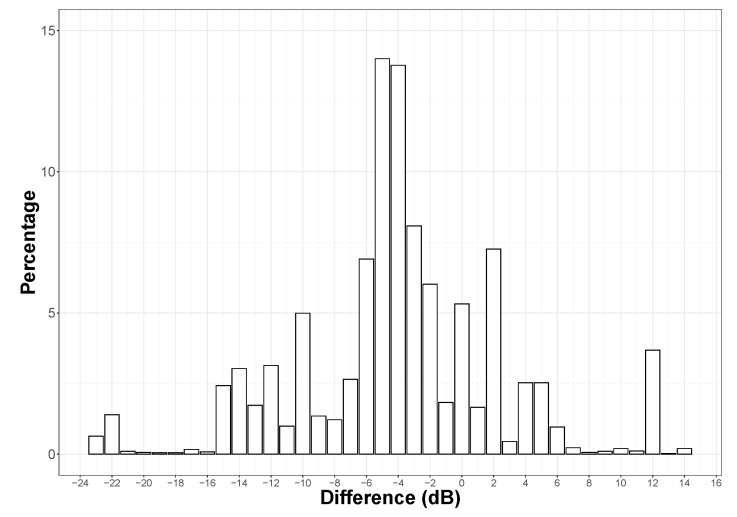
Distribution of the differences (in dB) of the noise level measured in each hexagonal zone, between the initial calibrated noise map and the noise map after blind calibration with the hybrid model, for the whole City of Rezé (for smartphones that were initially calibrated only). Differences are calculated for each hexagonal area (15 m) that composed the City of Rezé. *Y*-axis is given in terms of a percentage (%) of hexagonal area characterized by a given difference in dB.

In addition, we now consider the application of the hybrid method on the City of Rezé, with a minimum number of links of 100, which corresponds to the best configuration for the full dataset. As an illustration, Figure 12 shows the comparison between calibrated noise maps, either by considering the individual smartphone calibration values (as measured on the smartphone), or by considering the calibration values obtained using the hybrid blind calibration method, for a small part of the City of Rezé:

- The noise map (in dBA) produced with the initial calibration values (‘Initial’ noise map, Figure 12a). It considers only data for smartphones with an initial calibration (134 pairs).
- The noise map (in dBA) obtained by applying the blind calibration, using the hybrid method with a minimum threshold of 100 links per smartphone, but only for the smartphones that were initially calibrated (‘Blind calibrated’ noise map, Figure 12b). In this case, 52.7% of smartphones were calibrated (54 using the NGM method and 53 using the SMM method), enabling 71.9% of measurement points to be corrected.
- The difference map (in dBA) between the Initial and the Blind calibrated noise maps (Figure 12c); this difference map is calculated on the basis of the differences in the sound level in each hexagon. This map is completed in Figure 13 by a representation of the distribution of sound level differences as a percentage of the total number of corresponding hexagons in the whole City of Rezé (8464 hexagons contain data on all 10,365 hexagons).

A qualitative comparison of the map produced using the calibration values initially entered by users and the map produced after blind calibration provides some first insights into the method. The initial map (Figure 12a) cannot completely serve as a reference, because there may be errors in the calibration values entered by users. On the other hand, the blind calibration method also allows a calibration value to be estimated for smartphones that have not been calibrated, which is an asset that is not evaluated here.

The findings are as follows. The blind calibration method tends to result in a noise map with higher noise values in this case study. It is probably due to a bias linked to the assumption that the calibration values are centered on zero, which is not necessarily the case for a small number of smartphones. In fact, of the 134 smartphones, 10 correspond to almost half of the measurements, and in this particular case, the average gain calibration given for these smartphones is negative and slightly overestimated by the method. This is visually accentuated in the neighbourhoods where few measurements contribute to the estimated value for each hexagon. This is the case for instance in the northwest where the density of measurements is small (see Figure 6 of Reference [16]).

That said, Figure 13 shows that the dispersion of the differences between the two maps is fairly small with a large part of the points concentrated between −8 and +2 dB, which confirms the validity of the method (this distribution would be probably centered for an input dataset whose calibration values are centered on zero). The average value of sound level differences in the hexagons is overall very close to the average value of differences between the initial calibration values and after the blind calibration mentioned in the previous Section 3.5.2 (−3.4 dB), which seems consistent.

Particular behaviors can be observed on this distribution, such as a peak at +12 dB. A detailed analysis of each of the blind calibration values obtained for each (smartphone, user) pair and an assessment of the individual contribution of each pair in each of the hexagonal zones would be required in order to make any assumptions about these peaks. This question could be the subject of a future investigation.

It will also be interesting, in a further study, to test the behavior of the method as a function of the input datasets in order to adapt it to the study areas; this point is discussed in the following section.

## 4. Conclusions

Mobile noise measurements offer an alternative way of producing noise maps and collecting data on the noise environment through a participatory approach in which every citizen can become a data producer. Over and above the interest in contributing to the evaluation and development of public policies, this project raises real research questions, particularly in relation to the quality of the data produced and its use in an operational or regulatory context. Recent work on NoiseCapture data has shown a certain heterogeneity in the data collected, for example, in the absence of acoustic calibration of smartphones, a lack of expertise in the field of environmental acoustics by the contributors, or difficulties in implementing a measurement protocol that could be shared by all contributors. Data cleaning and quality control are therefore essential stages in the relevant use of the information collected. The work presented in this article is part of this approach and was aimed more specifically at implementing a generic calibration method for all data simultaneously. It is now accepted that it will never be possible to calibrate each smartphone individually and that a mass calibration should therefore be considered instead.

Among the solutions envisaged, those based on blind calibration approaches, already tested on other studies such as for air quality measurements, are an interesting perspective. In the present article, we have exploited a method that takes advantage of the multiple rendezvous of several smartphones “at the same place” and “at the same time”, measuring the same acoustic event. Written as a network graph model, the resolution of the associated matrix system can then be used to determine a mean drift for each smartphone, which is similar to a calibration correction in acoustics. The method relies on certain constraints, which are discussed in the paper, such as the temporal distribution of the data at our disposal to verify the “at the same time” condition, or more accurately at similar periods in the day, as well as the size of the spatial area to verify the “at the same place” condition. In addition, the number of rendezvous a smartphone can have with others is an important factor for the quality of results. In particular, we proposed a hybrid approach to address this critical point, enabling us firstly to improve the quality of the calibration on a limited number of smartphones by using the Network Graph Model, then, secondly, using these calibrated smartphones to calibrate the other ones using a simpler approach. With regard to the first limitation, the progressive accumulation of new data over time should make it possible to obtain a more relevant temporal distribution of data. We have also observed that considering only smartphones with a minimum time of presence in each spatial area could be a way to enhance the behavior of the hybrid method. Regarding the second limitation related to the size of the spatial area, the results show that a 15 m radius spatial area was sufficient to verify a relatively homogeneous noise environment in the context of the hybrid method.

The obtained results seem particularly interesting and demonstrate the feasibility of such a blind calibration approach for mobile noise data. The method can also be improved by taking advantage of the simultaneous presence of reference sensors in a given area, such as noise observatories or calibrated smartphones, as suggested in [44].

The behavior of the method could also be studied on the basis of a perfectly controlled virtual mobile noise measurement dataset, as shown in Section 2.3.3. For example, it would be possible to study in more detail the effects of time of presence in hexagons, temporal and spatial variability, minimum number of links, or the presence of reference sensors. It could be useful to identify with more confidence the best conditions for applying the hybrid blind calibration method and to adapt its parameter values to the characteristics of the dataset. A virtual mobile noise measurement dataset will also enable testing other spatial and temporal grids, replacing for instance hexagons by streets with similar traffic behavior or refining the “at the same time” condition relying on temporal periods with similar sound levels. It will be of interest finally to test the sensibility of the method to datasets with different levels of heterogeneity in the participatory contributions, as this first analysis suggests that some main contributors might have an influence on the method if they collect a large proportion of the data and have calibration values not centered on zero.

More generally, to improve the method, it might also be useful to improve the quality of the data collected. It could be envisaged at the source, by improving the mobile application to ensure better control of the measurement procedure but also a better understanding of the measurement context. One example is the possibility offered by specific libraries for the development of smartphone applications to obtain information on the user mode of travel (on foot, by bike, on public transport), or even the location of the smartphone itself (in the hand, in the pocket, in a bag). Improving the quality of the database can also be achieved a posteriori by searching for and then removing any data collected that could be assimilated to anomalies. It can be taken into account, for example, by considering methods such as the Local Outlier Factor (LOF) [45] or the Isolation Forest [46] methods.

To conclude, the blind calibration approach, possibly considering improvements, is a very interesting way of tackling the difficulty of calibrating each individual smartphone. With this methodology and as part of a participative approach to noise map production, it would no longer be necessary to ask users to calibrate their smartphones, as this can be completed a posteriori. It would also be interesting if each user could know the calibration value in return and have it automatically integrated into the application parameters.

## Figures and Tables

**Table 1:** Co-location sensor measurements based on the scenario of Figure 2.

Smartphone∖Zone	$Z^{1}$	$Z^{2}$	$Z^{3}$	$Z^{4}$	$Z^{5}$
$S_{1}$		$x_{1}^{2}$	$x_{1}^{3}$	$x_{1}^{4}$	
$S_{2}$		$x_{2}^{2}$	$x_{2}^{3}$	$x_{2}^{4}$	
$S_{3}$	$x_{3}^{1}$		$x_{3}^{3}$		$x_{3}^{5}$
$S_{4}$	$x_{4}^{1}$	$x_{4}^{2}$			$x_{4}^{5}$

**Table 2:** Application of the NGM on NoiseCapture Parties datasets (reference data).

‘pk_party’	Country	Tracks	Points	Time Period (24-Hour Format)	Nb of Sensors	Nb of Cal. Sensors	Zones	Links	$l_{d}$
10	Italy	149	15,912	11:00–12:00	12	11	479	357	5.4
13	France	100	21,470	10:00–11:00	11	11	817	508	9.2
22	France	192	17,309	12:00–19:00	23	23	403	1902	7.5
26	Italy	332	23,220	10:00–12:00	20	20	619	2526	13.3

**Table 3:** Effect of the size of the hexagon on the hybrid method as a function of the minimum number of links per smartphone. When the minimum number of links is equal to 1, it corresponds to the reference NGM. Mean error and uncertainty are given in dB.

		Hexagon Size			
**Minimum Number of Links**		**10 m**	**15 m**	**30 m**	**50 m**
1 (NGM)	Mean error	−2.33	0.36	−0.97	−1.28
	Uncertainty	±7	±8	±7	±6.5
15	Mean error	−2.33	−0.04	−0.97	−1.21
	Uncertainty	±7	±7.5	±7	±6.5
40	Mean error	−2.77	−2.13	−3.12	−3.69
	Uncertainty	±6.5	±5.5	±7.5	±7
55	Mean error	−3.86	−2.77	−3.71	−2.54
	Uncertainty	±6	±5	±5	±5
80	Mean error	−3.37	−2.34	−3.72	
	Uncertainty	±5	±4	±4.5	
120	Mean error	−3.54	−1.88		
	Uncertainty	±4	±3.2		
140	Mean error	−3.48	−1.92		
	Uncertainty	±4	±2.5		
190	Mean error	−4.76			
	Uncertainty	±3.5			

**Table 4:** Errors between the gain calibration value of smartphone and the obtained drift value, as a function of the number of links, in terms of mean error, median error and interquartile range (IQR), for the full dataset and the filtered data.

Minimum Number of Links per Sensor	1	5	10	20	50	100	200	500
Full dataset								
IQR	19.4	18.6	18	16.2	11.4	6.8	21.1	26.6
Mean	−6	−6	−6	−5.4	−2.7	−3.4	−11.8	−12
Median	−6.5	−6.5	−6.5	−6	−2.5	−3.4	−12.2	−11.8
Number of (smartphone, user) pairs	201	169	155	145	94	72	37	30
Filtered dataset—10 s								
IQR	18.9	17.9	17.1	15.7	11.1	19.6	22.6	
Mean	−4.2	−5.1	−2.1	−4	−2.9	−3.6	−9.9	
Median	−3.7	2.8	−1.3	−4.9	−1.8	−4.7	−12.6	
Number of (smartphone, user) pairs	163	131	108	85	57	26	19	
Filtered dataset—20 s								
IQR	17	16.9	20.4					
Mean	−3.8	−3.6	−4.8					
Median	−2.9	−2.8	−5.5					
Number of (smartphone, user) pairs	101	45	18					
Filtered dataset—30 s								
IQR	16.3	18.9	19					
Mean	−3.1	−0.7	−3.6					
Median	−3.9	−1.5	−0.8					
Number of (smartphone, user) pairs	63	20	12					

## Data Availability

The data presented in this study for the 2017–2020 period are openly available from the Université Gustave Eiffel Dataverse Repository at https://doi.org/10.25578/J5DG3W (accessed on 13 July 2023).

## References

[1] European Parlamient Directive 2002/49/EC of the European Parliament and of the Council of 25 June 2002 relating to the assessment and management of environmental noise—Declaration by the Commission in the Conciliation Committee on the Directive relating to the assessment and management of environmental noise. *Off. J.*
**2022**, *L 189*. http://data.europa.eu/eli/dir/2002/49/oj/eng.

[2] Can A., Dekoninck L., Botteldooren D. (2014). Measurement network for urban noise assessment: Comparison of mobile measurements and spatial interpolation approaches. Appl. Acoust..

[3] Maisonneuve N., Stevens M., Ochab B. (2010). Participatory Noise Pollution Monitoring Using Mobile Phones. Inf. Polity.

[4] Kanjo E. (2010). NoiseSPY: A Real-Time Mobile Phone Platform for Urban Noise Monitoring and Mapping. Mob. Netw. Appl..

[5] D’Hondt E., Stevens M., Jacobs A. (2013). Participatory noise mapping works! An evaluation of participatory sensing as an alternative to standard techniques for environmental monitoring. Pervasive Mob. Comput..

[6] Guillaume G., Can A., Petit G., Fortin N., Palominos S., Gauvreau B., Bocher E., Picaut J. (2016). Noise mapping based on participative measurements. Noise Mapp..

[7] Brambilla G., Pedrielli F. (2020). Smartphone-Based Participatory Soundscape Mapping for a More Sustainable Acoustic Environment. Sustainability.

[8] Picaut J., Fortin N., Bocher E., Petit G., Aumond P., Guillaume G. (2019). An open-science crowdsourcing approach for producing community noise maps using smartphones. Build. Environ..

[9] Picaut J., Boumchich A., Bocher E., Fortin N., Petit G., Aumond P. (2021). A Smartphone-Based Crowd-Sourced Database for Environmental Noise Assessment. Int. J. Environ. Res. Public Health.

[10] Picaut J., Fortin N., Bocher E., Petit G. (2021). Université Gustave Eiffel Online Repository for Research Data. NoiseCapture Data Extraction from August 29, 2017 until August 28, 2020 (3 Years). https://data.univ-gustave-eiffel.fr/dataset.xhtml?persistentId=doi:10.25578/J5DG3W.

[11] Noise-Planet Website (2023). Exploit NoiseCapture Data. https://noise-planet.org/noisecapture_exploit_data.html.

[12] Noise-Planet Website (2022). NoiseCapture Privacy Policy. https://noise-planet.org/NoiseCapture_privacy_policy.html.

[13] Zipf L., Primack R.B., Rothendler M. (2020). Citizen scientists and university students monitor noise pollution in cities and protected areas with smartphones. PLoS ONE.

[14] Guillaume G., Aumond P., Bocher E., Can A., Ecotière D., Fortin N., Foy C., Gauvreau B., Petit G., Picaut J. (2022). NoiseCapture smartphone application as pedagogical support for education and public awareness. J. Acoust. Soc. Am..

[15] Lefevre B., Agarwal R., Issarny V., Mallet V. (2021). Mobile crowd-sensing as a resource for contextualized urban public policies: A study using three use cases on noise and soundscape monitoring. Cities Health.

[16] Can A., Audubert P., Aumond P., Geisler E., Guiu C., Lorino T., Rossa E. (2023). Framework for urban sound assessment at the city scale based on citizen action, with the smartphone application NoiseCapture as a lever for participation. Noise Mapp..

[17] Kardous C.A., Shaw P.B. (2014). Evaluation of smartphone sound measurement applications. J. Acoust. Soc. Am..

[18] Zhu Y., Li J., Liu L., Tham C.K. iCal: Intervention-free Calibration for Measuring Noise with Smartphones. Proceedings of the 2015 IEEE 21st International Conference on Parallel and Distributed Systems (ICPADS).

[19] Murphy E., King E.A. (2016). Testing the accuracy of smartphones and sound level meter applications for measuring environmental noise. Appl. Acoust..

[20] Ventura R., Mallet V., Issarny V., Raverdy P.G., Rebhi F. (2017). Evaluation and calibration of mobile phones for noise monitoring application. J. Acoust. Soc. Am..

[21] Nast D.R., Speer W.S., Prell C.G.L. (2014). Sound level measurements using smartphone “apps”: Useful or inaccurate?. Noise Health.

[22] Aumond P., Lavandier C., Ribeiro C., Boix E.G., Kambona K., D’Hondt E., Delaitre P. (2017). A study of the accuracy of mobile technology for measuring urban noise pollution in large scale participatory sensing campaigns. Appl. Acoust..

[23] Garg S., Lim K.M., Lee H.P. (2019). An averaging method for accurately calibrating smartphone microphones for environmental noise measurement. Appl. Acoust..

[24] Aumond P., Can A., Rey Gozalo G., Fortin N., Suárez E. (2020). Method for in situ acoustic calibration of smartphone-based sound measurement applications. Appl. Acoust..

[25] Kardous C.A., Shaw P.B. (2016). Evaluation of smartphone sound measurement applications (apps) using external microphones—A follow-up study. J. Acoust. Soc. Am..

[26] Roberts B., Kardous C., Neitzel R. (2016). Improving the accuracy of smart devices to measure noise exposure. J. Occup. Environ. Hyg..

[27] Celestina M., Hrovat J., Kardous C.A. (2018). Smartphone-based sound level measurement apps: Evaluation of compliance with international sound level meter standards. Appl. Acoust..

[28] Celestina M., Kardous C.A., Trost A. (2021). Smartphone-based sound level measurement apps: Evaluation of directional response. Appl. Acoust..

[29] Can A., Guillaume G., Picaut J. (2016). Cross-calibration of participatory sensor networks for environmental noise mapping. Appl. Acoust..

[30] Pődör A., Szabó S. (2021). Geo-tagged environmental noise measurement with smartphones: Accuracy and perspectives of crowdsourced mapping. Environ. Plan. Urban Anal. City Sci..

[31] Miluzzo E., Lane N.D., Campbell A.T., Olfati-Saber R., Nikoletseas S.E., Chlebus B.S., Johnson D.B., Krishnamachari B. (2008). CaliBree: A self-calibration system for mobile sensor networks. Distributed Computing in Sensor Systems.

[32] Wang C., Ramanathan P., Saluja K.K. Moments based blind calibration in mobile sensor networks. Proceedings of the 2008 IEEE International Conference on Communications.

[33] Wang C., Ramanathan P., Saluja K.K. Blindly Calibrating Mobile Sensors Using Piecewise Linear Functions. Proceedings of the 2009 6th Annual IEEE Communications Society Conference on Sensor, Mesh and Ad Hoc Communications and Networks (secon 2009).

[34] Lee B.T., Son S.C., Kang K. (2014). A Blind Calibration Scheme Exploiting Mutual Calibration Relationships for a Dense Mobile Sensor Network. IEEE Sens. J..

[35] Bocher E., Petit G., Picaut J., Fortin N., Guillaume G. (2017). Collaborative noise data collected from smartphones. Data Brief.

[36] NoiseCapture App (2017). NoiseCapture Privacy Policy (Short Version). https://github.com/Universite-Gustave-Eiffel/NoiseCapture/blob/master/app/src/main/assets/html/privacy_policy.html.

[37] Noise-Planet App (2021). Localization Policy. https://github.com/Universite-Gustave-Eiffel/NoiseCapture/blob/master/app/src/main/assets/html/localisation_notice.html.

[38] (2011). Open Data Commons Open Database License (ODbL). https://opendatacommons.org/licenses/odbl/.

[39] Whitehouse K., Culler D. Calibration as Parameter Estimation in Sensor Networks. Proceedings of the 1st ACM International Workshop on Wireless Sensor Networks and Applications.

[40] Brocolini L., Lavandier C., Quoy M., Ribeiro C.F. (2013). Measurements of acoustic environments for urban soundscapes: Choice of homogeneous periods, optimization of durations, and selection of indicators. J. Acoust. Soc. Am..

[41] Graziuso G., Grimaldi M., Mancini S., Quartieri J., Guarnaccia C. (2020). Crowdsourcing Data for the Elaboration of Noise Maps: A Methodological Proposal. J. Phys. Conf. Ser..

[42] Graziuso G., Mancini S., Francavilla A.B., Grimaldi M., Guarnaccia C. (2021). Geo-Crowdsourced Sound Level Data in Support of the Community Facilities Planning. A Methodological Proposal. Sustainability.

[43] Siliézar J., Aumond P., Can A., Chapron P., Péroche M. (2023). Case study on the audibility of siren-driven alert systems. Noise Mapp..

[44] Dorffer C., Puigt M., Delmaire G., Roussel G. (2018). Informed Nonnegative Matrix Factorization Methods for Mobile Sensor Network Calibration. IEEE Trans. Signal Inf. Process. Netw..

[45] Breunig M.M., Kriegel H.P., Ng R.T., Sander J. (2000). LOF: Identifying density-based local outliers. ACM SIGMOD Rec..

[46] Liu F.T., Ting K.M., Zhou Z.H. Isolation Forest. Proceedings of the 2008 Eighth IEEE International Conference on Data Mining.

